# Molecular prevalence of *Dirofilaria immitis* and *Wolbachia* infections in pet and semi-domesticated cats in Bangkok, Thailand

**DOI:** 10.14202/vetworld.2022.239-243

**Published:** 2022-02-03

**Authors:** Naris Thengchaisri, Tawin Inpankaew, Surapong Arthitwong, Jörg M. Steiner, Panpicha Sattasathuchana

**Affiliations:** 1 Department of Companion Animal Clinical Sciences, Faculty of Veterinary Medicine, Kasetsart University, Bangkok, Thailand; 2 Tippimarn Veterinary Hospital, Chulabhorn Royal Academy, 906/1 Pong Ta Long Subdistrict, Pak Chong district, Nakhon Ratchasima, 30130, Thailand; 3 Department of Parasitology, Faculty of Veterinary Medicine, Kasetsart University, Bangkok, Thailand; 4 Department of Anatomy, Faculty of Veterinary Medicine, Kasetsart University, Bangkok, Thailand; 5 Department of Small Animal Clinical Sciences, Gastrointestinal Laboratory, College of Veterinary Medicine and Biomedical Sciences, Texas A&M University, College Station, Texas, USA

**Keywords:** cat, heartworm, prevalence, *Wolbachia*

## Abstract

**Background and Aim:**

Although cats are not natural hosts for heartworm infections (*Dirofilaria immitis*), evidence suggests that feline heartworm disease can be detrimental because of a severe inflammatory response. Recent studies have found that infection with bacteria of the genus *Wolbachia* is the principal cause of acute inflammatory filaria disease; nonetheless, the prevalence of cats naturally infected with heartworms and *Wolbachia* remains unclear. This study aimed to estimate the prevalence and current distribution of feline heartworm disease and its association with *Wolbachia* infection in pet and semi-domesticated cats in Bangkok, Thailand.

**Materials and Methods:**

A total of 260 cats (130 pet cats and 130 semi-domesticated cats) were enrolled in this study. Blood samples were placed into ethylenediaminetetraacetic acid tubes for hematological analysis and DNA extraction. A polymerase chain reaction (PCR) was performed to analyze samples for the presence of *D. immitis* and *Wolbachia* infections.

**Results:**

The prevalence (95% confidence interval [CI]) of *D. immitis* infection in pet, semi-domesticated, and all cats were 3.9% (1.3-8.8%), 27.7% (20.2-36.2%), and 19.6% (15.0-25.0%), respectively. The prevalence (95% CI) of *Wolbachia* infection in pet, semi-domesticated, and all cats were 18.5% (12.2-26.2%), 31.5% (23.7-40.3%), and 25.0% (19.9-30.7%), respectively. The prevalence of *D. immitis* and *Wolbachia* infections in semi-domesticated cats was significantly higher than in pet cats (p=0.002 and p=0.022, respectively). There was a significant association between *D. immitis* and *Wolbachia* infections (p<0.001). There was also a significant association between *D. immitis* infection and the presence of eosinophilia (p<0.045).

**Conclusion:**

From the PCR analysis, it can be concluded that semi-domesticated cats were at higher risk for *D. immitis* infection than pet cats. There was a significant association between positive *D. immitis* infection and positive *Wolbachia* infection. Combinations of anthelmintic and antimicrobial therapy should be considered in heartworm-positive cats.

## Introduction

*Dirofilaria immitis* is a vector-borne parasite that causes cardiopulmonary diseases in dogs and cats worldwide [1-8]. Dogs are a major reservoir for *D. immitis*, but cats and humans have also been reported as incidental hosts [7]. Cats are considered naturally resistant hosts because of the high mortality rate of L5 larvae in the pulmonary arteries of cats [4,9]. In Thailand, the prevalence of *D. immitis* in pet and semi-domesticated cats has been reported as 4.6% and 36.4%, respectively [3,6]. Heartworm disease can lead to eosinophilic pneumonitis and severe pulmonary thromboembolism [10]. Heartworm-associated respiratory disease has been linked with the release of the endosymbiotic bacterium from filarial nematodes [11]. *Wolbachia* endosymbiont is a Gram-negative, intracellular bacterium harbored in all life stages of parasites [12]. *Wolbachia* organisms have been reported in filarial nematodes, such as *Brugia malayi* and *D. immitis* [13,14]. *Wolbachia* surface proteins and antibodies are released when the parasite dies [12,14]. Although the pathogenetic role of *Wolbachia* in mammalian host cells is not clearly identified, *Wolbachia* antigens and antibodies have been known to stimulate the inflammation of mammalian host cells [14,15].

Several reports have identified the prevalence of *D. immitis* in dogs. The prevalence of *Wolbachia* infection among cats in Italy and dogs in Thailand has been reported at 10.4% and 82.5%, respectively [16,17]. To the best of our knowledge, there has been no report concerning the prevalence of *Wolbachia* infection among cats in Thailand. In addition, there are limited reports of the prevalence of and the association between *D. immitis* and *Wolbachia* infection in cats. Cats are often raised in households as human companions; thus, zoonotic diseases from cats may be transmitted to humans. *D. immitis* and *Wolbachia* endosymbiont infections have been reported in humans [18,19].

The aims of this study were 3-fold: (1) Estimate the prevalence of *D. immitis* and *Wolbachia* endosymbiont infections; (2) evaluate the association between *D. immitis* and *Wolbachia* infections in pet and semi-domesticated cats; and (3) evaluate the association between *D. immitis* infection and hematological parameters.

## Materials and Methods

### Ethical approval and i nformed consent

All sample collection protocols were reviewed and approved by the Kasetsart University Institutional Animal Care and Use Committee (approval number #ACKU60-VET-032). The study adhered to the ARRIVE (Animal Research: Reporting of *In Vivo* Experiments) guidelines. All cat owners were informed about the study goals, their rights, participant requirements, and permission to collect a blood sample from their cat. Written informed consent for semi-domesticated cats and client-owned cats was obtained from the head of the community or the cat’s owner, respectively.

### Study period and location

The domesticated cat samples were collected from cats visiting Kasetsart University Veterinary Teaching Hospital, Bangkok, Thailand, for an annual check-up between July 2017 to June 2018. The semi-domesticated cats samples were collected from cats living in the Thai temples in Bangkok, Thailand, from March 2017 to June 2017. The samples were processed at Faculty of Veterinary Medicine, Kasetsart University, Bangkhen campus.

### Sample collection

A total of 260 cats were enrolled in the study: 130 pet cats and 130 semi-domesticated cats. A complete history was collected for all domesticated cats, and a physical examination was performed to enroll only clinically healthy cats with a history of routine flea control and deworming programs. A whole-blood sample of approximately 3 mL volume was collected by a licensed veterinarian through either a cephalic or jugular puncture and was placed into an ethylenediaminetetraacetic acid (EDTA)-anticoagulated blood tube. A complete blood count was immediately performed using an automated hematology analyzer (Abbott CELL-DYN 3700 Hematology Analyzer, Abbott, Germany). The EDTA blood samples were stored at −20°C before DNA extraction.

### DNA extraction

DNA was extracted from 250 μL of EDTA-anticoagulated blood using a commercial DNA extraction kit (E.Z.N.A.® Blood DNA Mini Kit, Omega Bio-Tek Inc., Norcross, GA, USA) according to the manufacturer’s instructions. Final elution of the DNA was made in 100 μL of Tris-EDTA buffer (10 mM Tris-Cl, 0.5 mM EDTA, pH 9.0). The extracted DNA was stored at −20°C until used for the polymerase chain reaction (PCR) assays.

### PCR analysis and sequencing

All extracted DNAs were subject to PCR assays as previously described [20] to detect *D. immitis* infection based on the cytochrome C oxidase subunit I (COI) gene. *Wolbachia* infection was detected based on the *Wolbachia* protein-coding housekeeping ftsZ gene [16], as shown in Table-1 [16,20]. The PCR product was analyzed using 1.5% agarose gel electrophoresis. All positive PCR products were purified using the FavorPrep™ GEL/PCR Purification Kit (Favorgen Biotech Corporation, Pin-Tung, Taiwan) according to the manufacturer’s protocol and submitted to Macrogen (Macrogen Inc., Seoul, Korea) for DNA sequencing. All sequences were accumulated using Finch TV 1.4.0 (Geospiza Inc., Seattle, WA, USA) and compared with known sequences from the GenBank™ database (National Center for Biotechnology Information, Bethesda, MD, USA) using the BLAST (Basic Local Alignment Search Tool) algorithm (http://blast.ncbi.nlm.nih.gov/Blast.cgi).

**Table 1:** Oligonucleotide primers used to detect *D.* *immitis* and *Wolbachia* endosymbiont in blood samples from cats.

Pathogen	Gene target (size [bp])	Oligonucleotide sequence	Annealing temperature	Reference
*D. immitis*	COI (200)	DI COI-F1 (5′- AGTGTAGAGGGTCAGCCTGAGTTA-3′) DI COI-R1 (5′- ACAGGCACTGACAATACCAAT-3′)	59°C	[20]
*Wolbachia* endosymbiont	*FtsZ* (147)	Wol1-fwd (5’- CCTGTACTATATCCAAGAATTACTG-3’) Wol1-R (5’- ACTATCCTTTATATGTTCCATAATTTC-3’) Wol7-fwd (5’- GGTGGAAATGCTGTGAATAAC-3’) Wol7-R (5’- AGCACCGAGCCCTTTAG-3’)	51°C 57°C	[16]

*D. immitis*=*Dirofilaria immitis*, COI=Cytochrome C oxidase subunit I

### Statistical analysis

All analyses were performed using commercially available statistical software packages (JMP version JMP Pro 10, SAS institute, Cary, NC, USA; GraphPad Prism version 5.0, Graph-Pad Software, LAJolla, CA, USA; and STATA version 14.2 StataCorp LLC, College Station, TX, USA), with p<0.05 defined as statistically significant. The prevalence and 95% confidence interval (CI) of *D. immitis* and *Wolbachia* infections in pet and semi-domesticated cats were determined using cross-tabulations. The prevalence of *D. immitis* and *Wolbachia* infections in pet and semi-domesticated cats was compared using Fisher’s exact test. Fisher’s exact test was also used to determine the association between *D. immitis* and *Wolbachia* infections, between *D. immitis* infection and hematological parameters, and between *Wolbachia* infection and hematological parameters.

## Results

The mean age±standard deviation (SD) and the age range of all cats were 4.1±2.9 years and 0.3-15 years, respectively. The mean age±SD and the minimum and maximum ages of the semi-domesticated cats were 2.8±2.0 years and 0.83-13 years, respectively. The sex distribution of pet and semi-domesticated cats was 62 males versus 68 females and 47 males versus 83 females. All semi-domesticated cats were domestic shorthair cats, whereas the pet cats consisted of 67 shorthair and 63 longhair cats. The nucleotide sequences of the partial COI gene for which the filarial nematodes were able to be classified was 98-100% identical with *D*. *immitis* (GenBank: MK250757). The sequences of the ftsZ gene for *Wolbachia* identified in cats were 100% identical to the *Wolbachia* endosymbiont of *D. immitis* (GenBank: AJ495000).

The prevalence (95% CI) of *D. immitis* infection in pet, semi-domesticated, and all cats was 3.9% (1.3-8.8%), 27.7% (20.2-36.2%), and 19.6% (15.0-25.0%), respectively (Table-2). The prevalence of *D. immitis* infection in semi-domesticated cats was significantly higher than in pet cats (p=0.002; Table-2). The prevalence (95% CI) of *Wolbachia* infection in pet, semi-domesticated, and all cats was 18.5% (12.2-26.2%), 31.5% (23.7-40.3%), and 25.0% (19.9-30.7%), respectively (Table-2).

**Table 2:** Summary of *D. immitis* and *Wolbachia* endosymbiont infections in pet and semi-domesticated cats.

Diagnosis	Total (n)	Positive (n)	Negative (n)	Prevalence (%; CI)	p-value
*D. immitis*					
Pet cats	130	15	115	3.85 (1.26-8.75)	
Semi-domesticated cats	130	36	94	27.69 (20.21-36.22)	
Total cats	260	51	209	19.62 (14.97-24.97)	0.002
*Wolbachia* endosymbiont					
Pet cats	130	24	106	18.46 (12.20-26.21)	
Semi-domesticated cats	130	41	89	31.54 (23.67-40.27)	
Total cats	260	65	195	25.00 (19.86-30.72)	0.022

*D. immitis*=*Dirofilaria immitis*, CI=Confidence interval

The prevalence of *Wolbachia* infection in semi-domesticated cats was significantly higher than in pet cats (p=0.022; Table-2). There was an association between *D. immitis* and *Wolbachia* infections (p<0.001; Table-3).

**Table 3:** Association between *D. immitis* and *Wolbachia* endosymbiont infections in all cats.

*D. immitis* infection	*Wolbachia* endosymbiont infection			p-value

	Positive (n)	Negative (n)	Total	
Positive	51	0	51	
Negative	14	195	209	
Total	65	195	260	<0.001

*D. immitis*=*Dirofilaria immitis*

There was no association between *D. immitis* infection and the presence of anemia (p=0.718) or leukocytosis (p=0.278; Table-4). However, there was a significant association between *D. immitis* infection and the presence of eosinophilia (p=0.045; Table-4). There was no association between *Wolbachia* infection and the presence of anemia (p=0.764), leukocytosis (p=0.364), or eosinophilia (p=0.089; Table-5).

**Table 4:** Association of hematological parameters and *D. immitis* infection in pet and semi-domesticated cats.

Hematological results	*D. immitis* infection		p-value

	Positive (n)	Negative (n)	
Anemia			
Yes	3	16	0.718
No	26	182	
Leukocytosis			
Yes	7	30	0.278
No	22	168	
Eosinophilia			
Yes	20	92	0.045
No	9	101	

*D. immitis*=*Dirofilaria immitis*

**Table 5:** Association of hematological parameters and *Wolbachia* infection in pet and semi-domesticated cats.

Hematologic results	Wolbachia infection		p-value

	Positive (n)	Negative (n)	
Anemia			
Yes	4	15	0.764
No	39	169	
Leukocytosis			
Yes	9	28	0.364
No	34	156	
Eosinophilia			
Yes	27	85	0.089
No	16	94	

## Discussion

Even though dogs are a natural host for heartworm infection, cats and other mammal species can also be affected. The present study found that the overall prevalence of *D. immitis* infection among cats in Bangkok was 19.6%. Although more than a quarter of semi-domesticated cats (27.7%) were affected with *D. immitis* infection, the prevalence of *D. immitis* infection in pet cats (3.9%) was significantly lower. The overall *Wolbachia* endosymbiont infection prevalence in Bangkok cats was 25.0%. The prevalence of *Wolbachia* endosymbiont infection in semi-domesticated cats (31.5%) was also significantly higher than in pet cats (18.5%). Cats with positive *D. immitis* infection had a significantly higher risk of having *Wolbachia* infection than cats negative for *D. immitis* infection. There was also an association between *D. immitis* infection and the presence of eosinophilia, suggesting the presence of host response to heartworm infection in cats.

Detection of the adult female *D. immitis* antigen is commonly used to detect heartworm infection in dogs. However, a false-negative *D. immitis* antigen test is more common in cats than dogs because cats are usually infected with a low number of mature heartworms; moreover, worms exhibit a short lifespan, and only female heartworms are detectable by the antigen test [20]. In the present study, PCR detection of microfilaria was used to identify heartworm infection. A PCR-based method provides highly sensitive and specific detection of heartworm infection, and this method has been demonstrated to identify occult heartworm infections in dogs [21]. Our PCR-based assay results suggest a higher prevalence of heartworm infection in semi-domesticated cats than in pet cats. These findings make sense, since semi-domesticated cats reside with dogs and live outdoors.

Although cats are atypical hosts for heartworms, our findings indicated a high prevalence of semi-domesticated cats positive for heartworms (27.7%). A lower prevalence of heartworm infection among pet cats (3.9%) was detected in this study. The discrepancy between the two cat populations may be because pet cats regularly receive anthelmintic medications, which may be combined with flea prevention products. Most pet cats live indoors with humans and are thus protected from mosquito bites. Moreover, semi-domesticated cats share their habitats with dogs, which are a major reservoir of *D. immitis*.

Doxycycline is recommended for dogs with heartworm infection to treat concurrent *Wolbachia* endosymbiont infection [13,22]. This study demonstrated a strong relationship between *D. immitis* and *Wolbachia* infections (p<0.001). Thus, antimicrobial drugs for the treatment of *Wolbachi*a infection should be considered along with anthelmintic drugs to treat heartworm infection in cats and dogs. The number of cats with positive *D. immitis* infection (n=51) was lower than those with positive *Wolbachia* infection (n=65). All cats positive for *D. immitis* were also positive for *Wolbachia* infection. This may be because the *Wolbachia* genus is an obligate intracellular bacteria identified in many nematodes such as *Dirofilari*a spp. and *Brugia* spp. [13,15]. *Wolbachia* infection identified in our study may be acquired from *D. immitis* and other nematode species. Moreover, a study recently reported *B. malayi* and *Wolbachia* endosymbiont infection in cats residing in Thailand [15]. It is possible that *B. malayi* may be a contributing factor to the result of cats being negative for *D. immitis* but positive for *Wolbachia* infection.

The hematological findings indicated that positive *D. immitis* infection is associated with the presence of eosinophilia. Eosinophils are an important contributor to eliminating nematode infestations through a type 2 immune response [23-25]. Heartworm disease in cats can also lead to the development of eosinophilic pneumonitis [17]. The release of the *Wolbachia* from filarial nematodes may elicit a host immune response by activating eosinophils, leading to the release of granule proteins. Moreover, the presence of anemia and leukocytosis was not associated with *D. immitis* or *Wolbachia* endosymbiont infection, which may be because the enrolled cats in this study were clinically healthy at the time of sample collection. Thus, this study found no relationship between abnormal hematological profiles (except eosinophilia) and infection with *D. immitis* or *Wolbachia*.

## Conclusion

From the PCR analysis, it can be concluded that there was a significant association between positive *D. immitis* infection and positive *Wolbachia* infection. Semi-domesticated cats were at higher risk from *D. immitis* and *Wolbachia* infection than pet cats. As our study was based on small sample size, further studies with larger sample size should be performed to determine the relationship between hematological abnormalities in cats with either *D*. *immitis* or *Wolbachia* infection. Non-antimicrobial compounds, such as antioxidants and antihistamines, may also prove useful to minimize the host immune response to filarial infection.

## Authors’ Contributions

NT and TI: Designed the study, performed the experimental work, and reviewed the manuscript. SA: Performed the study and reviewed the manuscript. JMS: Data interpretation, project advisor, and reviewed the manuscript. PS: Designed the study, conducted the literature review, performed the experimental work, interpreted the data, and drafted the manuscript. All authors read and approved the final manuscript.
